# A study on shelf life of prepackaged retail-ready Korean native black pork belly and shoulder butt slices during refrigerated display

**DOI:** 10.5713/ab.21.0172

**Published:** 2021-06-23

**Authors:** Van-Ba Hoa, Kuk-Hwan Seol, Sun-Moon Kang, Yun-Seok Kim, Soo-Hyun Cho

**Affiliations:** 1 Animal Products Utilization Division, National Institute of Animal Science, RDA, Wanju 55365, Korea; 2 R & D Performance Evaluation & Management Division, RDA, Jeonju 54875, Korea

**Keywords:** Acceptability, Color, Off-flavor, Pork Belly, Shelf Life

## Abstract

**Objective:**

In most retail centers, primal pork cuts for sale are usually prepared into retail-ready slices and overwrapped with air-permeable plastic film. Also, meat of Korean native black pig (KNP) is reputed for its superior quality, however, its shelf life during retail display has not been studied. Thus, the objective of this study was to evaluate shelf life of prepackaged retail-ready KNP belly and shoulder butt slices during refrigerated display.

**Methods:**

Bellies and shoulder butt obtained at 24 h post-mortem from finishing KNP were used. Each belly or shoulder butt was manually cut into 1.5 cm-thick slices. The slices in each cut type were randomly taken and placed on white foam tray (2 slices/tray) overwrapped with polyvinyl chloride film. The retail-ready packages were then placed in a retail display cabinet at 4°C. Shelf life and sensory quality of the samples were evaluated on day 1, 3, 6, 9, 12, and 15 of display.

**Results:**

The shoulder butt reached the upper limit (20 mg/100 g) of volatile basic nitrogen for fresh meat after 9 days while, the belly remained within this limit throughout the display time (15 days). Both the cuts reached a thiobarbituric acid reactive substances level of above 0.5 mg malondialdehyde/kg after 9 days. The a* (redness) values remained unchanged during first 9 days in both cuts (p>0.05). After 9 days, off-flavor was not found in either cut, but higher off-flavor intensity was found in shoulder butt after 12 days. The shoulder butt was unacceptable for overall eating quality after 12 days while, belly still was acceptable after 12 days.

**Conclusion:**

The belly showed a longer shelf life compared to the shoulder butt, and a shelf life of 9 and 12 days is recommended for the prepackaged retail-ready KNP shoulder butt and belly slices, respectively.

## INTRODUCTION

Basically, the main objective of packaging is to protect foodstuffs from contaminations with dirt, toxic substances, and microorganisms (e.g., bacteria and virus) during transport, distribution, and storage processes as well as to prevent spoilage and weight losses [1]. In retail meat centers, primal meat cuts (e.g., beef and pork etc.) are often cut into thin slices or pieces that afterward are packaged using different methods such as modified atmosphere-, overwrapped- and vacuum-packaging [2,3]. Amongst these, overwrap-packaging, where the meat pieces or slices are placed onto trays and over-wrapped with air-permeable plastic film, is one of the most predominantly used aerobic methods in the retail shops/butcheries [2–5]. These retail-ready and consumer-sized retail packages (already prepared as the final cut) are displayed in self-service refrigerated cases for sale [6]. Practically, this overwrap-packaging provides customers with ease of use and convenience, reduces time and labor costs (increased labor productivity) [7]. In many developed countries, therefore, the preparation of retail-ready meat packs could be considered as an economical and convenient solution [2].

It is well known that the anoxic packaging methods (e.g., vacuum-packaging) prolong the shelf life of meat but produces a purple-red color due to deoxymyglobin formation. However, consumers generally are less familiar with this non-attractive color in the retail centers [8]. Whereas, the overwrap-packaging allows a quick pigment oxygenation process and desirable cherry-red color development which is more attractive to consumers [9]. Additionally, this packaging method allows showing the product characteristics (e.g., lean color and amounts of fat) during daily display, which also facilitates consumers’ handling, selection and inspection [10].

Shelf life of meat is defined as a period of time (between packaging and end use) that the product retains its acceptability from sensory and microbiological perspectives [11]. During refrigerated storage, a series of undesirable changes such as; lipid oxidation, protein decomposition, discoloration, and growth of spoilage bacteria etc. may occur, that cause rancid- and off-flavors of meat [4]. Therefore, the onset of spoilage of meat can be defined as when certain bacteria reach maximum acceptable level and the meat develops off-flavor [12]. Early works have indicated that the deterioration of meat during storage is strongly affected by many factors such as; endogenous factors, packaging methods and conditions, and duration [2]. Though the overwrap-packaging is the most commonly used method in the retail meat centers due to its distinct advantages as above mentioned, it still shows some limitations on the shelf life stability of meat due to the O_2_ atmosphere which promotes the lipid oxidation and discoloration etc. [10]

In Korea, the meat of Korean native black pig (KNP) is reputed for its superior quality; indicated by a more reddish color, whiter fat, higher marbling degree and better eating quality than those from commercial pig breeds [13,14]. For decades, KNP meat has been considered as a premium and delicious variety regardless of its approximately 30% to 40% more expensive price compared to the other commercial pig breeds-derived meats [15]. Until now, there are some studies evaluating the shelf life stability of fresh pork tenderloin, leg and loin chops during retail display [16–18]. As is known, pork belly and shoulder butt are the two the most consumed and preferable cuts in many countries [19], they are commonly sold in the retail stores/butcheries and supermarkets in the form of retail-ready slices displayed on trays overwrapped with plastic film. However, no studies have been conducted to investigate the shelf life stability of commercial pork belly and shoulder butt in general and KNP meat in particular during retail display. Thus, the aim of this study was to evaluate the shelf life and sensory properties of prepackaged retail-ready KNP belly and shoulder butt slices during refrigerated display.

## MATERIALS AND METHODS

### Animal care

The animal protocols used in the present study were reviewed and approved by the Institutional Animal Care and Use Committee (IACUC) at National Institute of Animal Science (Approval No. NIAS 20001992).

### Meat samples and packaging

Pork bellies (about 50 kg) and shoulder butt (25 kg) obtained at 24 h post-mortem from KNP (n = 6) were used in the present investigation. The pigs (barrow) were raised in a same pen at the experimental farm (National Institute of Animal Science), and fed *ad libitum* with a same commercial diet until slaughter (average body weight 112 kg). After slaughtering and chilling for 24 h, the carcasses were transferred to a cutting room where they were fabricated according to the instruction of Korean Pork Cutting Specification [20]. The shoulder butt cuts were removed between the first cervical vertebra and 4th rib, and bellies were removed from the shoulder between the 4th thoracic vertebrae with a straight perpendicular cut to the axis of the carcass. After removing skin and ribs, the bellies and shoulder butts (n = 6 each) from left carcass sides were collected and used for investigation of shelf life stability during retail display.

**Figure 1:**
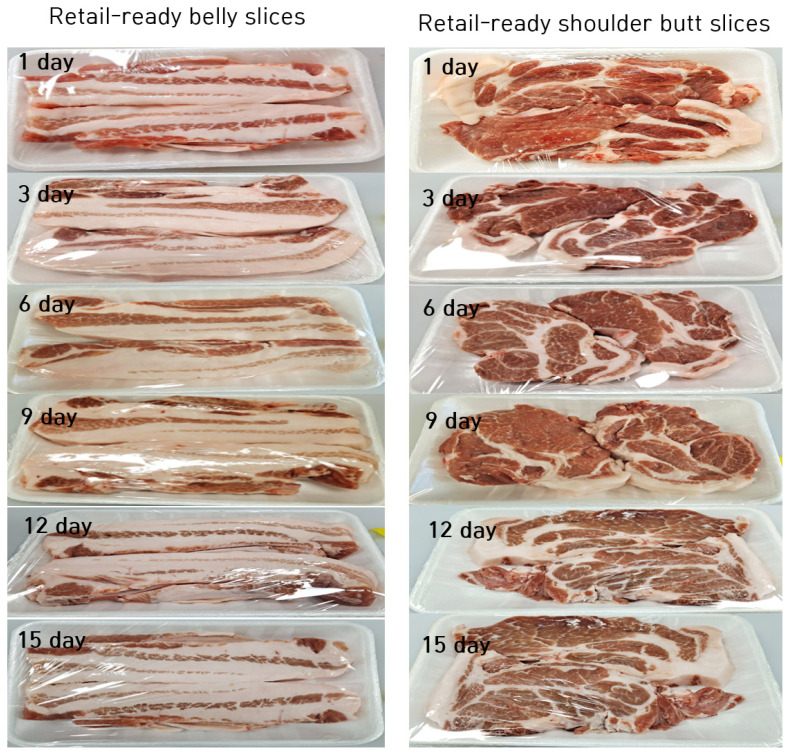
Representative images of prepackaged retail-ready belly and shoulder butt slices during refrigerated display at 4°C.

Each belly (only the 5th to last rib position was used) was manually cut (following the dorsal to ventral direction) into 18 slices with a thickness of about 1.5 cm. Similarly, each shoulder butt was cut into 12 slices with a thickness of about 1.5 cm. The cutting manner was similar to that used in the Korean meat retail centers/butcheries. Thereafter, all slices formed in bellies or shoulder butts were combined together and two slices were randomly taken and placed on white foam trays (27×18×2 cm) and overwrapped with 0.15 μm polyvinyl chloride film, resulting in 36 and 54 retail-ready trays for the shoulder butt and belly cut, respectively. Initial weights of slices in each tray were also recorded before overwrapping. The overwrapped meat trays were randomly divided into 6 equal portions (6 and 9 trays per portion for the shoulder butt and belly, respectively) that were allocated into 6 display periods (1, 3, 6, 9, 12, and 15 days). Following the packaging and assigning, the samples were immediately displayed for 1, 3, 6, 9, 12, and 15 d at 4°C in a retail display cabinet (Figure 1). After being displayed for the specified periods, the samples were analyzed for shelf life including microbiological load, drip loss, color, pH, lipid oxidation, volatile basic nitrogen (VBN) and sensory properties.

### Shelf life assessment

#### Instrumental color

At the end of each display period, the color (air-exposed upper surface) of shoulder butt and belly slices was measured using a using a Minolta Chroma Meter CR-400 with a D65 illuminant*C and 2° observer (Minolta Camera, Osaka, Japan). Prior to use, the device was calibrated using standard white tile. Color was measured through the packaging film at five different random locations on each the retail-ready pack of shoulder butt or belly. Care was taken to avoid measuring on the fat area. The color was described as CIE L* (lightness), a* (redness), and b* (yellowness).

#### Drip loss

The retail-ready meat trays were opened by removing the wrapping-film, and the slices were removed from the trays, slightly mopped with wiping papers to remove fluid and re-weighed. The drip loss was calculated as weight loss and expressed as a percentage relative to initial weight.

#### Microbiological analysis

In each of the retail-ready packs, one slice was used for microbiological analysis, pH, lipid oxidation and VBN, and the remaining one was used for sensory evaluation. Particularly, immediately after removing the wrapping-film, microbiological samples were taken on both surfaces (upper and lower sides) of each the slice. The sampling was done by firmly rubbing sterile sponge (pre-moistened with peptone solution) over a 25-cm^2^ surface area (5×5 cm) of selected region of each slice. Thus, at each of the display periods, 12 (6 slices×2 sides/slice) and 18 (9 slices×2 sides/slice) microbial samples from a 300 and 450-cm^2^ surface area were taken from the shoulder butt and belly samples, respectively. All the sponge samples were then placed into individual 15-mL tubes containing 10 mL peptone solution, sealed and used for bacterial analysis. After vortexing for 1 min, tenfold dilutions were prepared with the same peptone solution for each the sample. Aerobic plate counts (APC) were enumerated by plating 1 mL of diluted sample onto Petrifilm APCs (3M Health Care; St. Paul, MN, USA) that were then incubated at 37°C for 48 h in an incubator.

#### pH measurement

The pH change in samples during display was measured using a pH*K 21 (NWK-Technology GmbH, Kaufering, Germany) equipped with a stainless steel and solid-state probe. The pH values ware measured by inserting the probe deeply into the slices (the same slices used for microbiological analysis). The pH values of each slice were the average of the three readings.

#### Thiobarbituric acid reactive substances

The extent of lipid oxidation in the samples during display was determined by measuring the thiobarbituric acid reactive substances (TBARS) content using the procedure of Buege and Aust [21]. Briefly, approximately 5.0 g of each sample was homogenized with 15 mL distilled water, 50 μL saturated butylated hydroxyansole and 15 mL of thiobarbituric acid (0.02 M)/trichloroacetic acid (15% w/v) (TBA/TCA at 1:1 ratio) at 11,000×rpm for 15 s using an Ultra-Turrax T25B. The mixture was adjusted to 50 mL with the TBA/TCA solution and immediately placed on ice. Thereafter, the samples were heated at 90°C in a water bath for 15 min. After cooling on ice for 20 min, the samples were centrifuged at 3,000×g for 10 min. About 1.5 mL of supernatant was taken and read at 531 nm using an UV–visible spectrophotometer (ProteomeLab Du-800, Beckman Coulter, Inc., Brea, CA, USA). TBARS values were calculated from a standard curve of 1,1,3,3 tetraetoxipropane, following the same procedure. The results were expressed as mg malondialdehyde/kg (MDA/kg) of meat. Lipid oxidation was measured in duplicate from each of the packages for each the cut type.

#### Volatile basic nitrogen

The VBN content was measured following the Conway method as described by Seong et al [22] with minor modifications. Briefly, after chopping and grinding, each sample (5.0 g) was placed into a test tube which was then homogenized with 45 mL of distilled water, and filtered through Whatman filter paper (No.1). Approximately 1 mL filtrate (from each sample) was taken and placed into the outer space of the Conway tool, and then 1 mL of 0.01 N H_3_BO_3_ and 100 μL of Conway reagent (0.066% methyl red: 0.066% bromocresol green, 1:1) were also added to the inner space. After that, 1 mL of 50% K_2_CO_3_ was added to the outer space of the Conway tool and was sealed immediately. The sealed Conway tool was incubated at 37°C for 2 h in an incubator. After incubating, various volumes of 0.01 N H_2_SO_4_ solution were added to the inner space until color changed to violet. The content was calculated and expressed as mg VBN/100 g meat.

### Visual color measurement and sensory quality evaluation

The visual meat color was evaluated at 3-days interval (1, 3, 6, 9, 12, and 15 d) of retail display. The surface color of the samples was evaluated using trained panelists (n = 7, who were staff at the institution) following the meat color guidelines set by American Meat Science Association [23]. Immediately after removing from the retail display cabinet, the retail-ready trays were evaluated by the panelists for the color using 7-point scale (7 = extremely like, 6 = like very much, 5 = like moderately, 4 = slightly like, 3 = dislike moderately, 2 = dislike very much, and 1 = dislike extremely). The mid-point of this scale (rating 3.5) was considered as the lowest score at which the panelists would purchase the product.

During evaluation, six testing sessions were carried out, each session evaluated 10 samples and each sample was evaluated by 7 panelists. The samples (entire slices) assigned in each session were directly placed on pre-heat open tin-coated grill for approximately 4 min and turned at the start of browning. The cooking temperature was monitored using an infrared thermometer and was maintained at around 160°C to 170°C. One set of grill was used where one slice was cooked each time. Immediately after cooking, each cooked slice was cut into 7 strips (30×30×15 mm) using a stainless steel food crocodile tong and scissor, the strips were then placed on individual paper dishes and served to the panelists. The panelists tasted and then evaluated for the sensory traits using a 7-point scale as described by Meilgaard et al [24]. For tenderness (1 = very tough and 7 = very tender), flavor (1 = dislike extremely and 7 = like extremely), juiciness (1 = very dry and 7 = very juicy), off-flavor (intensity of flavor as unappreciated for and overall acceptability; 1 = very week and 7 = very strong) and overall acceptability (1 = extremely unacceptable, 3.5 = between acceptable and unacceptable and 7 = extremely acceptable). The panelists were provided with drinking water and unsalted crackers to refresh their palate between samples. All sensory sessions were performed in the sensory panel booth room equipped with white lighting.

### Statistical analysis

The Statistical Analysis System (SAS) Enterprise 7.1 package (SAS Institute, Cary, NC, USA, 2018) was applied for analyzing the obtained data. Means and standard errors were calculated for the variables. The data was analyzed by using the General Linear Model procedure considering cut type and display time as the main effects. Means were compared using Duncan’s Multiple Range Test. Significance was defined at p<0.05. Correlation coefficients between the variables and display time were determined by using the Pearson’s linear correlation test. Principal component analysis (PCA) was also determined to explore relationships between variables and retail display time for the both shoulder butt and belly using the XLSTAT program 2020.3 (Addinsoft Inc., NY, USA).

## RESULTS AND DISCUSSION

The proximate composition of shoulder butt and belly samples studied were also determined and we found that the crude protein and fat contents were 16.04% and 27.99%, and 14.24% and 37.39% for the shoulder butt and belly, respectively.

### The changes in drip loss and APC during retail display

Drip loss is considered as an important technological quality trait because an excessive drip loss from fresh meat indicates not only financial losses but also losses in valuable nutrients, which reduce eating quality of meat [25]. In both cut types, the drip loss generally increased with increased display time, with a higher level (2.24%) in the shoulder butt compared to the belly (0.70%) after 15 days (Table 1). Compared to drip loss levels (13%) reported by Callejas-Cárdenas et al [26] for lamb steaks packaged using the same method at 14-day storage, all the samples in the present study had a lower level. Furthermore, on most examining days, the shoulder butt samples presented a significantly higher drip loss compared to those of the belly samples (p<0.05). This may be due to the difference in chemical composition such as fat content among the meat and cut types since the fat is negatively correlated to the moisture content in meat [27]. The result of Pearson correlation test also showed a positive correlation between display time and drip loss in the shoulder butt samples (r = 0.977, p<0.05) as shown in Table 2.

In general, the APC in all the samples significantly (p<0.05) increased with increased display time. However, the number of aerobic bacteria in all the samples were relatively low on all examining days; after 1 day, the APC were 0.26 and 0.14 log_10_ CFU/cm^2^ in shoulder butt and belly respectively. At the end of display (day 15), the APC only reached about 2.27 and 2.09 log_10_ CFU/cm^2^ in the shoulder butt and belly, respectively. Compared to our results, those of Custódio et al [16] and Li et al [18] found much higher bacterial counts; approximately 4 and 8 log_10_ CFU/g in pork loin and leg overwrapped with the same packaging material after 1 and 9 days of storage, respectively. Though the APC in all the samples increased with increased display time, the increasing rate seemed to be quite slow compared to that reported in these studies. This may be related to i) a low initial surface contamination which might be due to the guaranteed slaughter and fabrication hygiene and ii) a low drip loss (exudative fluid) level, because the exudative fluids contain a huge amount of nutrients that can favor bacterial growth.

### The changes in pH, TBARS, and VBN contents during retail display

The pH, TBARS, and VBN contents in the samples during display are presented in Table 3. The pH is considered as a reference indicator for meat freshness [28]. On day 1, the pH values were 5.78 and 5.71 for the shoulder butt and belly, respectively, and these values fell within the range (below 6.0) for normal post-rigor pork [29]. The display time resulted in an increase in the pH values for both the cut types; the shoulder butt and belly remained their pH values of below 6.0 after 6 and 9 days of display, respectively. The increase in meat pH during storage has been attributed to the basic chemical compounds (e.g., ammonia and amines etc.) that are produced from the degradative processes of peptides and amino acids by endogenous and microbial enzymes [28]. On the other hand, on all the examining days, the shoulder butt samples presented significantly (p<0.05) higher pH values compared to the belly samples. This might be related to the differences in the post-mortem muscle glycogen content and metabolisms between the cut types. Correspondingly, the result of Pearson correlation test also showed a positive correlation between the display time and pH in both the shoulder butt (r = 0.903, p<0.05) and belly (r = 0.857, p<0.05) samples (Table 2). This finding agrees with those of Li et al [18] and Custódio et al [16], who also showed a positive link between pH and storage time for overwrap-packaged fresh pork loin shops.

The VBN content is mainly composed of volatile primary, secondary, and tertiary derivatives of ammonia (e.g., amines) that are formed from the degradative processes of proteins and other nitrogen (N)-containing compounds in meat by spoilage mechanisms (e.g., endogenous and bacterial spoilage) during storage [30]. The VBN content is toxic and the cause of off-flavor which negatively affects the eating quality and acceptability of meat [28]. Therefore, total VBN content has commonly been used and considered as the most important indicator of freshness of meat [17]. Our results showed that the total VBN content in all the samples significantly increased with increased display time. On day 1, the VBN content was 4.94 and 3.37 mg/100 g in the shoulder butt and belly samples, respectively. At the end of display (day 15), the VBN content was 30.11 and 11.80 mg/100 g in the shoulder butt and belly samples, respectively. Thus, during the entire display time, the VBN content increased by 8.43 and 25.17 mg/100 g in the belly and shoulder butt samples, respectively. Our results align with those of Custódio et al [16] and Fan et al [17], who reported a similar trend for the VBN content in overwrap-packaged pork loin, leg and tenderloin samples during refrigerated storage. However, compared to our data, these authors reported a higher VBN level (over 60 mg/100 g) for the samples after 9 days of storage. These contrasting results could be attributed to the differences in the cut types (that differ in protein content), and the initial surface contamination of the samples among the studies. On the other hand, although the VBN content increased with increased display time, the increasing rate was considerably faster in the shoulder butt compared to the belly. This is probably related to the higher bacterial count (Table 1), protein content (16.04%, data not shown) which is the main source for the production of VBN, and activity of endogenous proteases in the shoulder butt samples. According the guideline for freshness by Korea Food and Drug Administration [31], a VBN level of 20 mg per 100 g is the upper limit for fresh meat. Thus, based on the obtained VBN results, it may be said that the overwrap-packaged retail-ready KNP shoulder butt and belly slices could remain fresh up to 9 and 15 days of display at 4°C, respectively.

Lipid oxidation is known as the major cause of quality loss and it results in development of off-flavors in meat and meat products [32]. Additionally, the lipid oxidation-derived secondary products can also react with proteins, resulting to protein oxidation and discoloration of meat [29]. Results showed that the TBARS values remained unchanged during the first 3 and 6 days in shoulder butt and belly, respectively (p>0.05). Within first 3 days, the TBARS values (0.31 to 0.32 mg MDA/kg) in the shoulder butt did not increase but it increased from day 6 to 15 (0.73 mg MDA/kg). For the belly, the TBARS values did not increase within first 6 days (0.32, 0.34, and 0.40 mg MDA/kg on day 1, 3, and 6, respectively), but it increased from day 9 to 15 (1.20 mg MDA/kg). Significant (p<0.05) difference between the shoulder butt and belly in TBARS values only was observed on day 15 of display. The result of Pearson correlation test also showed a positive correlation between display time and TBARS values in the shoulder butt (r = 0.914, p<0.05) and belly samples (r = 0.809, p<0.05) (Table 2). In agreement with our results, studies have reported an increase in TBARS values in overwrap-packaged pork and lamb as increasing storage time [5,17]. However, compared to our data, much higher TBARS values were reported for the samples in these studies. It has been found that the autoxidation, photo-oxidation and enzymatic hydrolysis are the major three lipid oxidation pathways in meat and meat products [32]. Amongst, enzymatic hydrolysis results from the action of enzymes (e.g., lipases and lipoxygenases) that are released from spoilages bacteria and muscle tissues [28]. In this present study, both the cut types studied were obtained from the same slaughter batch, and were packaged and displayed under identical conditions. Therefore, the higher TBARS level observed in the belly could be partly related to the higher activity of the endogenous enzymes (e.g. lipases and lipoxygenases) in this cut type. On the other hand, it has been reported that the TBARS level of above 0.5 mg MDA/kg in meat products indicates the off-flavors that can be detected by consumers [33]. Thus, both the cut types studied reached this limit after days 9 of display.

### The changes in visual and instrumental color

Consumers usually use color as the most important indicator of freshness and wholesomeness of meat [34]. Therefore, the color of fresh meat is a primary factor affecting the selection of meat at the point of purchase [29]. The results of visual and instrumental measurements for the retail-ready shoulder butt and belly samples during display at 4°C are presented in Table 4. Regarding the visual measurement, within the first 3 days no differences in rating scores occurred for the shoulder butt samples (p>0.05), but it was significantly (p<0.05) decreased from day 6 to day 15. For the belly samples, within the first 9 days no differences in the rating scores were reported (p>0.05), and the lower scores were only observed on the samples displayed for 12 to 15 days. Since the mid-point of this scale (rating 3.5) is considered as the lowest score at which the panelists would purchase the product, the retail-ready shoulder butt and belly reached this limit after 9 and 12 days, respectively. Thus, it may be said that the belly samples could remain their attractive color longer compared to the shoulder butt samples.

Regarding the instrumental color measurement, the display time had a significant effect on all color traits. For the shoulder butt, the L* (lightness) values significantly (p<0.05) increased during the first 3 days and then remained unchanged until day 12 and decreased thereafter. For the belly samples, the L* values remained unchanged during the first 9 days (p>0.05), and then increased thereafter (p<0.05). For the a* (redness), its changing trend during the retail display was opposite to that of the L* values. The a* values remained unchanged during the first 6 and 9 days for the belly and shoulder butt samples, respectively (p>0.05). And it significantly decreased from day 9 and 12 in the belly and shoulder butt samples, respectively (p<0.05). Similar to the L*, the b* (yellowness) values significantly (p<0.05) increased during the first 3 days and then remained unchanged for several days and significantly decreased after 12 to 15 days in both cut types. The correlation analysis also revealed a negative relationship between display time and visual color score (r = −0.894 and −0.894, p<0.05 in shoulder butt and belly, respectively) and a* value (r = −0.944 and −0.882, p<0.05) in shoulder butt and belly, respectively) (Table 2). In agreement with our results, studies have reported a similar changing trend of the L*, a*, and b* attributes in different prepackaged meat types (pork and lamb) in air-permeable packaging film [5,17]. The changes in visual and quantitative color traits observed on the samples during the display may be attributed to the oxidation of lipid and protein, and the lipid-protein oxidation interactions that resulted in the formation of metmyoglobin [28,35]. A negative correlation was also found between the TBARS and visual color score in the shoulder butt (r = −0.960, p<0.05) and belly samples (r = −0.874, p<0.05) (data not shown).

### The changes in sensory properties

The mean scores for the sensory attributes of the meat samples during display are presented in Table 5. For the flavor, no differences in scores were found for the shoulder butt or belly samples during the first 6 days (p>0.05). From day 9 to 15, the flavor scores were decreased in both cut types studied (p<0.05). Within the first 9 days, the panelists reported no differences in flavor scores between two cut types (p>0.05), but they gave higher scores for the belly than for shoulder butt after 12 to 15 days (p<0.05). Off-flavor was not found in both cut types within the first 9 days. After 12 days, the off-flavor scores significantly increased in all the samples, with higher intensity in shoulder butt compared to belly samples (p<0.05). Thus, after 12 to 15 days the higher flavor scores given for the belly samples may be related to their lower off-flavor scores (p<0.05). In agreement with our results, several studies have reported that extending storage time leads to increased off-flavor intensity in prepackaged meat [5]. The display time showed a minor effect on the tenderness; the panelists reported no differences in scores among the samples displayed for 1, 3, 6, 9, and 12 days (p>0.05). For the juiciness, within the first 12 days, the panelists also reported no differences in scores in shoulder butt samples (p>0.05), and significant (p<0.05) lower scores only were found on the 15-day displayed samples. Similar to the tenderness, higher juiciness scores were given for the belly samples on all the examining days (p<0.05). This may be attributed to the higher fat content in belly samples compared to shoulder butt as mentioned above. Regarding the overall acceptability, within the first 9 days, no differences in the rating scores were found for the shoulder butt or belly samples (p>0.05). In both shoulder butt and belly studied, the overall acceptability scores were decreased as extending the display time to 12 to 15 days (p<0.05). However, after 12 to 15 days, higher acceptability scores were found in the belly samples than in the shoulder butt (p<0.05). On the 7-point scale, the mid-point of this scale (rating 3.5) is considered as between the acceptable and unacceptable score, meaning that a rating score of above 3.5 is the lowest limit of acceptability. Thus, the shoulder butt samples were unacceptable after 12 days of display. Whereas, at the end of display (day 15), the belly samples still showed an acceptability score of 3.92. This indicates that the retail-ready belly slices were slightly acceptable from sensory quality after 15 days of display. The results of correlation test also showed a negative relationship between display time and flavor (r = −0.913, p<0.05), tenderness (r = −0.818, p<0.05), juiciness (r = −0.944, p<0.05) and acceptability (r = −0.892, p<0.05) in the shoulder butt samples (Table 2). Consistent with our results, several studies have also reported a decrease in sensory attributes score in prepackaged pork and lamb meat as increasing storage time [5,18].

#### Principal components analysis

**Figure 2:**
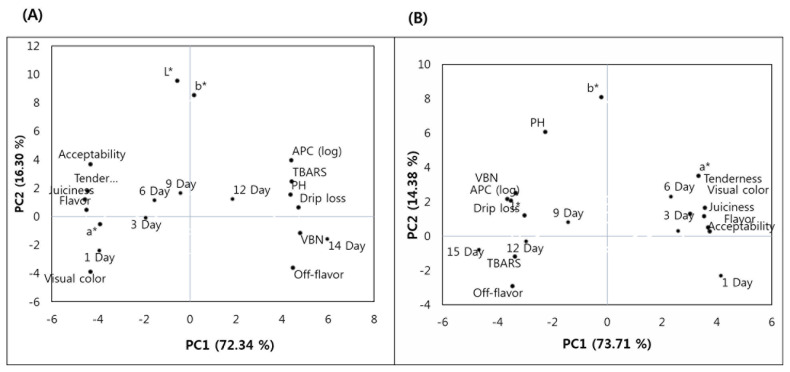
Principal component (PC) analysis for meat quality traits and shelf-life of retail-ready shoulder butt and belly cuts. (A) projection of variables and retail display time (1, 3, 6, 9, 12, and 15 d) for belly in plane defined by PC1 and PC2; (B) projection of variables and retail display time (1, 3, 6, 9, 12, and 15 d) for shoulder butt in plane defined by PC1 and PC2.

To obtain a trend of relationship between the observations and variables, and between the variables, PCA was carried out, and the results are shown in Figure 2. The PCA showed that about 72.34% and 16.30%, and 73.71% and 14.38% of variability were explained by the Principal components (PC)1 and PC2 for belly (Figure 2A) and shoulder butt (Figure 2B), respectively. For the belly, the samples displayed for the first 9 days (1, 3, 6, and 9 d) were on the negative PC1 axis, therefore, they were related to a*, tenderness, visual color, juiciness, flavor and acceptability. Whereas, the samples displayed for 12 to 15 days were on the positive PC1 axis, therefore, they were related to APC, TBARS, pH, drip loss, and VBN and off-flavor, L* and b*. Unlike the belly samples, only the shoulder butt samples displayed for the first 6 days (1, 3, and 6 d) were on the positive PC1 axis, therefore, they were related to a*, tenderness, visual color, juiciness, flavor and acceptability. Whereas, the shoulder butt samples displayed on day 9, 12, and 15 were on the negative PC1 axis, therefore, they were related to b*, L*, APC, TBARS, pH, drip loss, VBN, and off-flavor. Thus, the result of multivariate analysis by the PCA indicated a difference in shelf life stability between two cut types studied. In general, these observations were similar to the results presented in Table 1, 3, 4, and 5. Also, the trends of relationship were also observed in the Pearson correlation test results (Table 2).

## CONCLUSION

This study for the first time, evaluated the shelf life of prepackaged retail-ready KNP shoulder butt and belly slices during refrigerated display up to 15 days. The shoulder butt and belly samples remained the pH values of below 6.0 after 6 and 9 days, respectively. The shoulder butt reached the upper limit (20 mg VBN/100 g) of VBN for fresh meat after 9 days, while the belly remained within this limit entire the display time. Both the cut types reached a TBARS value of above 0.5 mg MDA/kg after 9 days of display. A decrease in a* (redness) values was observed in both cut types after 9 days of display. The results of visual color measurement showed that both shoulder butt and belly were reduced in their rating scores to 3.5 (the lowest limit at which the panelists would purchase the meat product) after 9 and 12 days of display, respectively. After 9 days of display, the off-flavor was not found in both cut types. From day 12 to the end of display, the off-flavor significantly increased, with higher intensity in the shoulder butt compared to the belly samples. The shoulder butt samples were unacceptable from overall sensory quality after 12 days whereas, the belly samples still were slightly acceptable after 15 days of display. Thus, a shelf life of 9 and 12 days is suggested for the prepackaged retail-ready KNP shoulder butt and belly slices in air-permeable packaging film, respectively.

## Figures and Tables

**Table 1:** Drip loss and aerobic plate count of retail-ready Korean native black pig shoulder butt and belly slices during refrigerated display

Display time (d)	Drip loss (%)		APC (log_10_ CFU/cm^2^)	
	
	Shoulder butt	Belly	Belly	Shoulder butt
1	0.72±0.21^b^^A^	0.39±0.12^b^^B^	0.14±0.13^f^	0.26±0.15^f^
3	0.98±0.56^b^	0.39±0.16^b^	0.69±0.13^e^	0.77±0.27^e^
6	1.35±0.86^ab^	0.60±0.25^ab^	1.10±0.23^d^	1.33±0.16^d^
9	1.28±0.54^ab^^A^	0.63±0.11^ab^^B^	1.49±0.19^c^	1.66±0.14^c^
12	1.56±0.54^ab^^A^	0.70±0.20^a^^B^	1.76±0.22^b^^B^	1.95±0.12^b^^A^
15	2.24±1.27^a^^A^	0.70±0.28^a^^B^	2.09±0.12^a^^B^	2.27±0.12^a^^A^

APC, aerobic plate count; CFU, colony-forming unit.

a–f Means in a column are significantly different (p<0.05).

A,B Means in a row within each analytic parameter are significantly different (p<0.05).

**Table 2:** Correlation coefficients between variables (quality parameters) and display time for retail-ready pork shoulder butt and belly slices

Variables	Shoulder butt	Belly
APC	0.909*	0.964*
TBARS	0.914*	0.890*
pH	0.903*	0.857*
Drip loss	0.977*	0.588
VBN	0.988*	0.932*
Visual color	−0.894*	−0.944*
Juiciness	−0.944*	0.396
Off-flavor	0.926*	0.521
Tenderness	−0.818*	0.499
Acceptability	−0.892*	−0.756*
Flavor	−0.931*	0.390
L*	−0.115	−0.797
a*	−0.807*	−0.882*
b*	0.036	−0.057

APC, Aerobic plate count; TBARS, thiobarbituric acid reactive substances; VBN, volatile basic nitrogen.

* Significant correlation (p<0.05).

**Table 3:** pH, VBN, and TBARS contents of retail-ready Korean native black pig shoulder butt and belly slices during refrigerated display

Display time (d)	pH		VBN (mg/100 g)		TBARS (mg MDA/kg)	
		
	Shoulder butt	Belly	Shoulder butt	Belly	Shoulder butt	Belly
1	5.78±0.08^d^^A^	5.71±0.07^A^^b^	4.94±0.92^e^^A^	3.37±0.89^d^^B^	0.31±0.01^d^	0.32±0.03^d^
3	5.92±0.10^cd^^A^	5.80±0.06^B^^dc^	9.55±1.53^d^^A^	5.39±0.90^c^^B^	0.32±0.00^d^	0.34±0.03^d^
6	5.97±0.16^bc^	5.82±0.28^c^	14.49±2.14^c^^A^	6.74±1.29^bc^^B^	0.44±0.10^c^	0.40±0.07^d^
9	6.02±0.27^bc^^A^	5.90±0.18^b^^B^	16.74±2.33^c^^A^	9.66±0.92^bc^^B^	0.53±0.11^b^	0.56±0.17^c^
12	6.12±0.15^b^	6.01±0.15^b^	22.69±0.76^b^^A^	10.56±1.12^ab^^B^	0.61±0.13^b^	0.69±0.12^b^
15	6.27±0.29^a^	6.13±0.14^a^	30.11±1.28^a^^A^	11.80±0.89^a^^B^	0.73±0.15^a^^B^	1.20±0.33^a^^A^

VBN, volatile basic nitrogen; TBARS, thiobarbituric acid re active substances; MDA, malondialdehyde.

a–e Means in a column are significantly different (p<0.05).

A,B Means in a row within each analytic parameter are significantly different (p<0.05).

**Table 4:** Visual and instrumental color of retail-ready Korean native black pig shoulder butt and belly slices during refrigerated display

Display time (d)	Visual color^1)^		L* (Lightness)		a* (Redness)		b* (Yellowness)	
			
	Shoulder butt	Belly	Shoulder butt	Belly	Shoulder butt	Belly	Shoulder butt	Belly
1	5.00±0.91^a^	5.10±0.88^a^	46.46±4.32^b^^B^	54.00±6.11^b^^A^	13.48±1.52^a^	12.65±1.02^a^	6.23±0.98^d^	6.99±1.50^d^
3	4.83±0.79^ab^	4.83±0.91^a^	51.35±2.80^a^^B^	55.50±3.77^b^^A^	12.74±1.27^a^	12.67±1.62^a^	9.30±1.82^b^^B^	11.05±1.29^a^^A^
6	4.43±0.94^b^	4.80±0.76^ab^	51.00±1.53^a^^B^	55.29±2.11^b^^A^	12.90±1.00^a^	12.66±0.90^a^	10.42±1.27^a^^B^	11.23±2.00^a^^A^
9	4.47±0.78^b^	4.60±0.67^ab^	50.78±2.57^a^^B^	57.14±4.97^b^^A^	12.29±1.39^a^^A^	10.21±1.54^b^^B^	10.92±0.92^a^	10.22±1.53^ab^
12	3.44±0.82^c^	3.64±0.86^c^	53.25±4.76^a^^B^	61.03±5.24^a^^A^	10.11±1.42^b^^A^	7.87±0.33^c^^B^	9.21±1.69^b^	9.25±1.87^bc^
15	3.12±1.05^c^	3.24±0.83^c^	45.92±3.06^b^^B^	61.09±5.57^a^^A^	9.57±1.17^b^^A^	6.96±0.77^c^^B^	8.00±1.03^c^	8.38±0.90^c^

1) Visual color evaluated by panelists using 7-point scale: 1 = extremely dislike, 7 = extremely like and mid-point (rating 3.5) = the lowest score at which the panelists would purchase the product.

a–d Means in a column are significantly different (p<0.05).

A,B Means in a row within each analytic parameter are significantly different (p<0.05).

**Table 5:** Mean scores (7-point scale) of sensory attributes of retail-ready Korean native black shoulder butt and belly slices during refrigerated display

Display time (d)	Flavor		Tenderness		Juiciness		Off-flavor		Acceptability	
				
	Shoulder butt	Belly	Shoulder butt	Belly	Shoulder butt	Belly	Shoulder butt	Belly	Shoulder butt	Belly
1	4.74±1.18^a^	5.21±1.26^a^	4.83±1.05^a^^B^	5.73±1.05^a^^A^	5.03±1.13^a^^B^	6.03±1.00^a^^A^	1.67±0.71^b^	1.50±0.68^b^	4.60±1.30^a^^B^	5.37±1.13^a^^A^
3	4.63±1.18^a^	4.90±0.93^ab^	4.70±1.02^ab^^B^	5.30±1.09^ab^^A^	4.97±0.81^a^^B^	5.63±1.16^ab^^A^	1.40±0.62^b^	1.40±0.56^b^	4.63±1.03^a^	5.13±1.22^ab^
6	4.40±1.27^a^	4.90±0.93^ab^	4.53±1.11^bc^^B^	5.50±1.07^bc^^A^	4.90±1.06^a^^B^	5.73±1.05^ab^^A^	1.57±0.68^b^	1.40±0.62^b^	4.37±0.89^a^^B^	5.20±1.19^ab^^A^
9	4.40±0.73^b^	4.47±0.81^bc^	4.63±0.67^bc^^B^	5.00±0.69^bc^^A^	4.67±0.76^a^^B^	5.23±0.82^bc^^A^	1.80±0.34^b^	1.80±0.81^b^	4.53±0.63^a^	4.77±0.82^bc^
12	3.48±0.91^c^^B^	4.20±0.71^c^^A^	4.20±0.65^bc^^B^	5.16±0.69^bc^^A^	4.24±0.60^ab^^B^	5.28±0.84^bc^^A^	3.00±1.29^a^^A^	2.20±0.96^a^^B^	3.46±0.82^b^^B^	4.44±0.96^cd^^A^
15	2.84±0.79^c^^B^	3.96±0.80^c^^A^	4.12±0.97^c^^B^	4.92±0.81^c^^A^	3.68±0.95^c^^B^	5.00±1.04^c^^A^	3.28±1.24^a^^A^	2.56±0.87^a^^B^	2.80±0.65^c^^B^	3.92±0.76^d^^A^

a–d Means in a column are significantly different (p<0.05).

A,B Means in a row within each sensory trait are significantly different (p<0.05).
